# Comparison of Survival Outcomes of Different Treatment Options for cT1-2, N0 Glottic Carcinoma: A Propensity Score–Weighted Analysis

**DOI:** 10.3389/fsurg.2022.902817

**Published:** 2022-05-20

**Authors:** Qi-wei Liang, Liang Peng, Jing Liao, Chun-xia Huang, Wei-ping Wen, Wei Sun

**Affiliations:** ^1^ Department of Otorhinolaryngology Head and Neck Surgery, Department of Thyroid Center/Thyroid Surgery, The Sixth Affiliated Hospital of Sun Yat-sen University, Guangzhou, China; ^2^ Department of Otorhinolaryngology Head and Neck Surgery, The First Affiliated Hospital, Sun Yat-sen University, Guangzhou, China

**Keywords:** glottic carcinoma, survival, inverse probability of treatment weighting, propensity score, treatment

## Abstract

**Background:**

Treatments for cT1-2, N0 glottic squamous cell carcinoma (GLSCC) include endoscopic resection, open surgery, and radiotherapy. The purpose of this study was to compare the outcomes of three treatment modalities and provide reference data for treatment selection.

**Methods:**

In all, 4274 patients with cT1-2, N0 GLSCC underwent these three treatment modalities from 2004 to 2015 were identified from the Surveillance, Epidemiology, and End Results-18 database. Overall survival (OS) and disease-specific survival (DSS) of patients treated with the three modalities were compared.

**Results:**

In the entire cohort, there were no significant differences in 5-year OS and 5-year DSS among the three treatment groups. In subgroup analyses based on stage and age, endoscopic resection provided significantly better 5-year survival than radiotherapy for cT1, N0 patients aged <65 years, with an OS rate of 89.0% vs. 82.3% (*p* = 0.009) and a DSS rate of 95.6% vs. 88.2% (*p* = 0.021). For 5-year DSS, open surgery also had better outcomes than patients who received radiotherapy (5-year DSS: 98.5% vs. 88.2%, respectively; *p* = 0.046).

**Conclusions:**

To summarize, for cT1, N0 GLSCC patients younger than 65 years, surgical treatment (either endoscopic or open) appears to be superior to the radiotherapy, and endoscopic resection should probably be the first consideration.

## Introduction

Laryngeal cancer is the second most common malignant tumor of the upper respiratory tract. There were 12,410 new cases of laryngeal cancer reported in the US in 2019, and about 3,760 patients died. The most common form of laryngeal cancer is glottic squamous cell carcinoma (GLSCC) (1). Patients with cT1-2, N0 (stage I and II) GLSCC should be treated to achieve cure as much as possible while preserving laryngeal function.. As multiple treatment modalities will increase the incidence of side effects and complications, cT1-2, N0 GLSCC is usually treated with a single modality. In the 2020 NCCN guidelines, open surgery or endoscopic resection, or radiotherapy are recommended (2), but it is not known whether the survival outcomes vary with these different treatments. While several studies found no significant differences in 5-year overall survival (OS) and disease-specific survival (DSS) with endoscopic resection versus radiotherapy (3–6), Chung et al. reported that local control rate and 5-year disease free survival were better with radiotherapy than with endoscopic resection (7); In view of the controversial survival outcomes of these studies, the choice of treatment to maximize benefits for patients with cT1-2, N0 is a clinical question worth thinking about.

Using the Surveillance, Epidemiology, and End Results (SEER)-18 database, we analyzed data from 4274 early-stage GLSCC patients to determine the 5-year OS and 5-year DSS achieved with each treatment using propensity score–weighted analysis to account for baseline differences. These findings may guide treatment selection for patients with cT1-2, N0 GLSCC and therefore improve survival rates.

## Materials and Methods

### Data Collection

The patient data were extracted from the SEER-18 database, using SEER*Stat 8.3.6.1 software. Patients who met the eligibility criteria were (1) they had been diagnosed between 2004 to 2015; (2) the lesion was in the glottis (anatomic site code C32.0 according to the International Classification of Diseases for Oncology, 3rd edition); (3) the histological type was squamous cell carcinoma, and (4) the disease was classified as stage I or II (i.e., T1N0M0 or T2N0M0, respectively, according to NCCN classification). We classified and analyzed the extracted data by age; sex; race; histologic grade; diagnosis year; tumor, node, metastasis (TNM) classification; American Joint Committee on Cancer (AJCC 6th edition) stage; marital status; and the type of treatment offered (endoscopic resection only, open surgery only, radiotherapy only).

### Statistical Analysis

The selected patients were divided into three groups based on the type of treatment they would recieve (endoscopic resection, open surgery group, or radiotherapy). Propensity score–weighted analysis was performed to balance for differences in potential cofounding factors between the groups. The general boosted model was used for estimating the multiple treatment propensity scores, which were adjusted by inverse probability of treatment weighting (IPTW) (8). The baseline characteristics that needed to be balanced included age, sex, race, diagnosis year, histologic grade, AJCC stage, and marital status. The balance of baseline characteristics was evaluated in pairs using absolute standardized deviation (ASD), with ASD <0.2 considered to indicate an acceptable balance. The propensity score weighting, balance assessment, and treatment effects estimation were performed in R 4.0.2 (http://www.r-project.org/). Calculations of survival rates were conducted using the Kaplan–Meier method and a log-rank test was performed to compare survival curves between unweighted and weighted cohorts.. The IPTW-adjusted hazard ratio (HR) was estimated with a Cox proportional hazards model. Two-tailed *p* < 0.05 was considered statistically significant.

## Results

### Baseline Patient Characteristics

**Figure 1:**
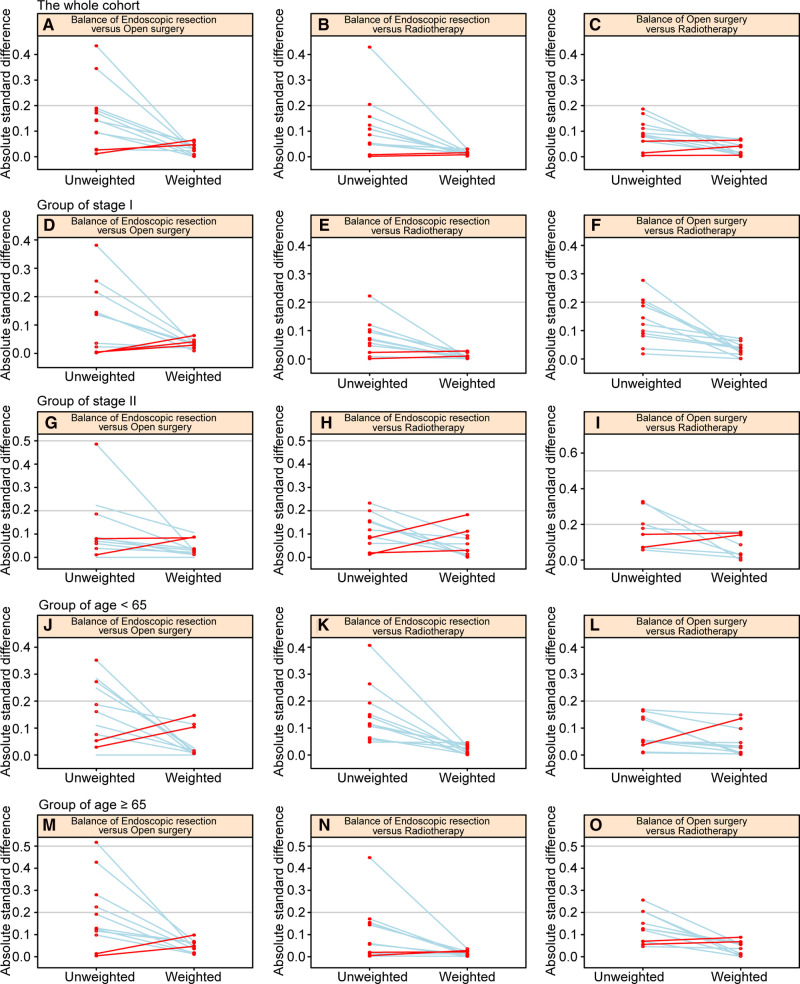
Paired graphs show the balance of baseline characteristics among treatment groups in the whole cohort (**A–C**), patients with stage I disease (**D–F**), patients with stage II disease (**G–I**), patients aged <65 years (**J–L**), and patients aged ≥65 years (**M–O**).

There were 4274 patients identified from the SEER database: 74.2% were treated with radiotherapy (*n* = 3172), 19.8% with endoscopic therapy (*n* = 845), and 6.0% with open surgery (*n* = 257) (Supplementary Table S1). Patients’ baseline characteristics are shown in Table 1 before and after IPTW adjustments. Figures 1A–C shows a paired-balance assessment of the three treatment groups. Using IPTW has achieved an excellent balance of baseline characteristics.

**Table 1:** Baseline characteristics in the three treatment groups before and after propensity score weighting.

Characteristic	Unweighted (%)				IPTW (%)			
	Endoscopic resection	Open surgery	Radiotherapy	ASD	Endoscopic resection	Open surgery	Radiotherapy	ASD
Race								
White	87.7	86.8	84.6	0.086	86.1	87.8	85.4	0.065
Black	8.2	8.6	12.2	0.124	11.1	9.0	11.2	0.068
Other	4.1	4.7	3.2	0.079	2.8	3.2	3.4	0.031
Age, years								
<65	45.6	54.1	45.6	0.171	46.6	46.1	46.2	0.009
≥65	54.4	45.9	54.4	0.171	53.4	53.9	53.8	0.009
Sex								
Male	87.5	91.8	89.0	0.141	89.3	91.1	88.9	0.07
Female	12.5	8.2	11.0	0.141	10.7	8.9	11.1	0.07
Diagnosis year								
2004–2009	39.9	47.1	50.1	**0** **.** **205**	47	47.2	48.0	0.019
2010–2015	60.1	52.9	49.9	**0**.**205**	53	52.8	52.0	0.019
Marital status								
Married	68.3	63.8	63.1	0.108	64.7	66.2	64.2	0.042
Single	31.7	36.2	36.9	0.108	35.3	33.8	35.8	0.042
Grade								
I	33.8	18.7	26.9	**0**.**345**	28.4	27.3	27.7	0.024
II	57.8	66.9	63.0	0.19	62.1	64.2	62.3	0.044
III	8.2	13.6	9.8	0.181	9.3	8.3	9.7	0.045
IV	0.2	0.8	0.3	0.096	0.2	0.2	0.3	0.017
Stage								
I	91.1	71.6	71.8	**0**.**434**	76.9	75.8	75.6	0.03
II	8.9	28.4	28.2	**0**.**434**	23.1	24.2	24.4	0.03

*IPTW, inverse probability of treatment weighting; ASD, absolute standardized difference.*

*ASD values in bold font indicate inadequate balance.*

### Survival Analysis

#### Entire Cohort

**Figure 2:**
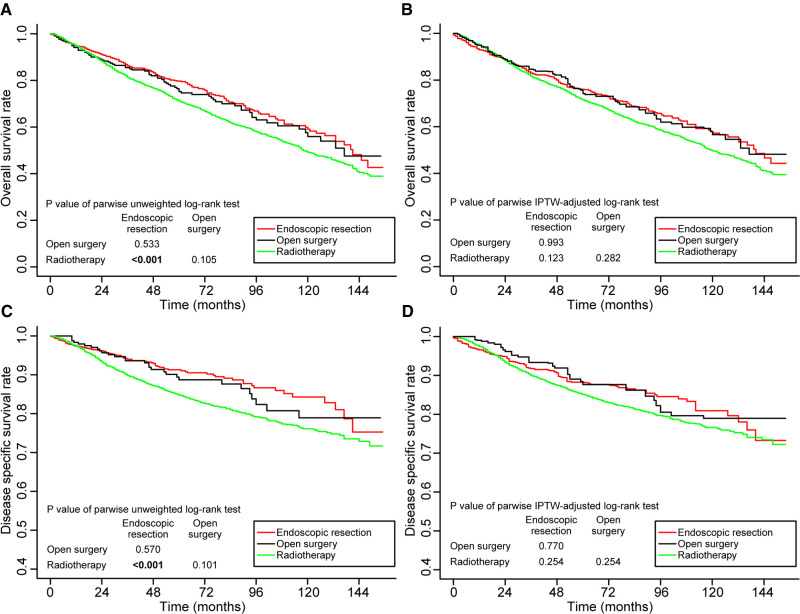
Kaplan–Meier curves for overall survival (**A,B**) and disease-specific survival (**C,D**) before and after weighting in the whole cohort. Significant p values are in bold font. IPTW, inverse probability of treatment weighting.

In the entire cohort, prior to adjustment for IPTW, the 5-year OS rates were better in the endoscopic resection group (79.7%, *p* < 0.001) and the open surgery group (75.2%, *p* = 0.105) compared to radiotherapy group (71.1%). Endoscopic resection versus open surgery did not differ statistically significantly (*p* = 0.533) (Figure 2A). After adjustment for IPTW, the 5-year OS rates of endoscopic resection, open surgery, and radiotherapy were 76.0%, 74.2%, and 71.6%, respectively. No statistically significant differences were found between the groups (Figure 2B).

Similarly, before IPTW adjustment, the 5-year DSS was better in the endoscopic resection group (91.3%, *p* < 0.001) and the open surgery group (88.7%, *p* = 0.101) than in the radiotherapy group (84.7%). Endoscopic resection versus open surgery did not show a statistically significant difference (*p* = 0.570; Figure 2C). The 5-year DSS rates after IPTW adjustment were 88.3%, 87.6%, and 85.1%, respectively, in the endoscopic resection, open surgery, and radiotherapy groups. It was not statistically significant to differentiate the groups (Figure 2D).

#### Subgroup Analysis

An analysis of subgroups based on disease stage was conducted. In stage I, there were 3,232 patients, while in stage II, there were 1,042 patients. Each subgroup was weighted by propensity scores. In Supplemental Tables S2 and S3, we summarize baseline characteristics of stage I and stage II patients before and after IPTW adjustment. The IPTW adjustment succeeded in balancing the baseline characteristics of patients, as shown in Figures 1D–I.

**Figure 3:**
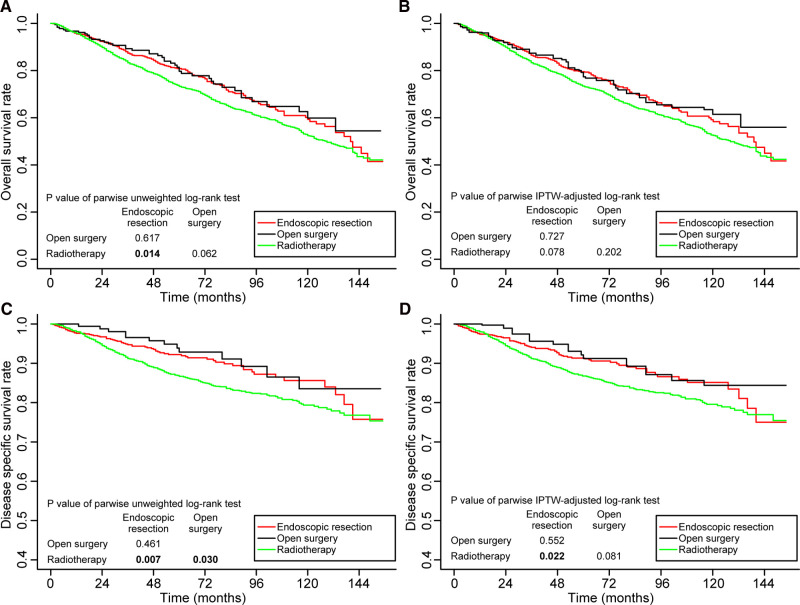
Kaplan–Meier curves for overall survival (**A,B**) and disease-specific survival (**C,D**) before and after weighting in patients with stage I disease. Significant p values are in bold font. IPTW, inverse probability of treatment weighting.

The survival outcomes of patients in stage I are shown in Figure 3. Prior to IPTW adjustment, endoscopic resection (80.9%, *p* = 0.014) and open surgery (79.7%, *p* = 0.062) had better 5-year OS rates than radiotherapy (73.3%). The difference between the OS rates achieved with endoscopic resection and open surgery was not found to be significantly different (*p* = 0.481) (Figure 3A). Following the IPTW adjustment, the 5-year OS rates in patients receiving endoscopic resection, open surgery, and radiotherapy were 79.5%, 77.4%, and 73.4% respectively. The differences between the three groups were not statistically significant (Figure 3B).

Meanwhile, before IPTW adjustment, the 5-year DSS was significantly better in patients receiving endoscopic resection (92.2%, *p* = 0.007) and open surgery (92.9%, *p* = 0.022) than in patients receiving radiotherapy (86.7%) (Figure 3C). Even after IPTW adjustment, the 5-year DSS remained better in patients receiving endoscopic resection (91.3%, *p* = 0.003) and open surgery (91.2%, *p* = 0.081) than those receiving radiotherapy (85.8%). The difference between patients who underwent endoscopic resection and those who underwent open surgery was not significant (*p* = 0.552) (Figure 3D).

Supplementary Figure S1 illustrates the survival outcomes of patients in stage II. There were no statistically significant differences found among OS and DSS at 5 years for patients treated with endoscopic resection, open surgery, or radiotherapy before or after IPTW adjustment.

To define the elderly, we used 65 years as the cutoff point (9), and divided the entire cohort into two age subgroups. There were 1972 patients aged <65 years and 2302 patients aged ≥65 years. There were 1972 patients aged <65 years and 2302 patients aged ≥65 years. Supplementary Tables S4 and S5 summarize the baseline characteristics of patients in the two age groups presented before and after IPTW adjustment. The IPTW adjustment achieved excellent balance in baseline characteristics, as illustrated in Figures 1J–O.

**Figure 4:**
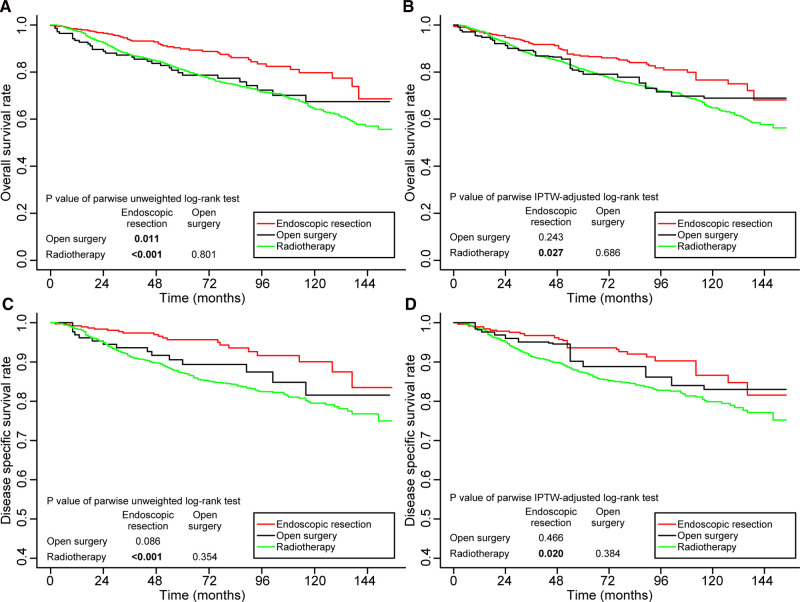
Kaplan–Meier curves for overall survival (**A,B**) and disease-specific survival (**C,D**) before and after weighting in patients aged <65 years. Significant p values are in bold font. IPTW, inverse probability of treatment weighting.

Figure 4 shows the survival outcomes of patients <65 years of age. The 5-year OS and 5-year DSS rates in patients receiving endoscopic resection were significantly higher than those in patients receiving radiotherapy, both before and after IPTW adjustment. Compared to open surgery, endoscopic resection had a significantly better 5-year OS rate (*p* = 0.011) before IPTW adjustment (Figure 4A), and the difference did not persist after IPTW adjustment (*p* = 0.466) (Figure 4B). Supplementary Figure S2 shows the survival outcomes of patients aged ≥65 years. In terms of OS and DSS, no significant differences were observed between the three treatment groups.

**Figure 5:**
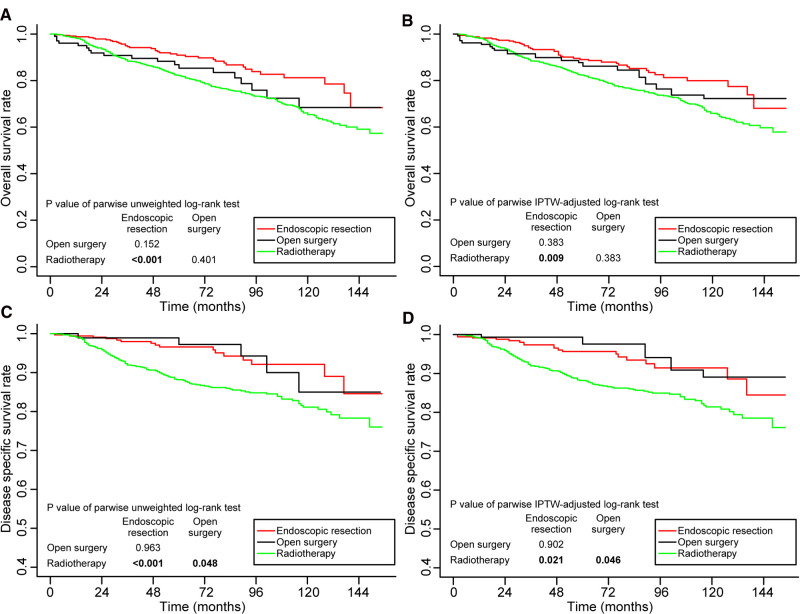
Kaplan–Meier curves for overall survival (**A,B**) and disease-specific survival (**C,D**) before and after weighting in the patients aged <65 years and in stage I. Significant p values are in bold font. IPTW, inverse probability of treatment weighting.

The cohort was subdivided into four subgroups to clarify the impact of age and stage on treatment outcomes: (1) patients aged <65 in stage I, (2) patients aged ≥65 in stage I, (3) patients aged <65 in stage II, and (4) patients aged ≥65 in stage II. We weighted the four subgroups based on propensity scores. In Supplementary Tables S6–S9, we summarize the baseline characteristics of patients in the four subgroups before and after IPTW adjustment. Supplementary Figures S3A–I illustrates the subgroups’ assessment of balance. Among patients aged <65 in stage I, both before and after IPTW adjustment, the 5-year DSS rates of patients treated with endoscopic resection and open surgery were significantly higher than those of patients treated with radiotherapy. Meanwhile, the 5-year DSS rate of patients treated with open surgery radiotherapy was significantly higher than that of patients assisted with radiotherapy options. The rates were similar in patients treated with open surgery and endoscopic resection (Figure 5). The OS and DSS rates achieved by patients in the other three groups did not differ significantly (Supplementary Figures S4–S6).

Table 2 displays the results of Cox proportional hazards regression analysis for the weighted whole cohort and the subgroups. The hazard ratio (HR) and 95% confidence intervals (CI) were consistent with the results of the log-rank tests.

**Table 2:** Cox proportional hazards regression analysis.

Group	OS		DSS	
	HR (95% CI)	p value	HR (95% CI)	p value
Whole cohort				
Endoscopic resection	Reference	Reference	Reference	Reference
Open surgery	1.02 (0.76–1.36)	0.900	0.96 (0.60–1.55)	0.879
Radiotherapy	1.20 (1.01–1.42)	**0** **.** **036**	1.27 (0.96–1.68)	0.094
Stage I				
Endoscopic resection	Reference	Reference	Reference	Reference
Open surgery	0.94 (0.64–1.39)	0.773	0.99 (0.47–2.09)	0.983
Radiotherapy	1.21 (1.03–1.43)	**0**.**018**	1.40 (1.06–1.85)	**0**.**015**
Stage II				
Endoscopic resection	Reference	Reference	Reference	Reference
Open surgery	1.12 (0.62–2.02)	0.694	0.99 (0.45–2.17)	0.978
Radiotherapy	1.13 (0.70–1.83)	0.611	1.01 (0.55–1.86)	0.960
Age <65 years				
Endoscopic resection	Reference	Reference	Reference	Reference
Open surgery	1.45 (0.86–2.48)	0.161	1.35 (0.59–3.10)	0.470
Radiotherapy	1.58 (1.12–2.25)	**0**.**008**	1.82 (1.10–3.05)	**0**.**020**
Age ≥65 years				
Endoscopic resection	Reference	Reference	Reference	Reference
Open surgery	0.85 (0.56–1.27)	0.431	0.78 (0.41–1.49)	0.467
Radiotherapy	1.08 (0.88–1.34)	0.434	1.09 (0.77–1.53)	0.624

*CI, confidence interval; HR, hazard ratio; OS, overall survival; DSS, disease-specific survival.*

*Significant p values are in bold font.*

## Disscussion

Our study compared the survival outcomes in a large cohort of 4274 cT1-2, N0 GLSCC patients treated by three different modalities: endoscopic resection, open surgery, and radiotherapy. The baseline characteristics of the three groups were balanced by propensity score weighting to improve the credibility of the results (10). Using the traditional covariable adjustment method based on regression, unmatched patients were excluded from the analysis, which reduced the generalizability and accuracy of the results. It is possible to overcome the disadvantage mentioned above by using the propensity score-weighted method. Using this method, all patients could be included in the analysis, and the impact of covariables on survival outcomes was well eliminated, making the effectiveness of different treatments comparable (10–12). Studies have shown that surgical treatment (either endoscopic or open) is superior to radiotherapy for cT1, N0 GLSCC patients younger than 65 years old, and that endoscopic resection is the most appropriate treatment option.

Currently, The most common treatment modalities for cT1-2, N0 glottic cancer are endoscopic resection and radiotherapy (13). Though open surgery is less frequently used due to limitations such as the long hospital stay and poor voice quality after treatment (14, 15), it is still a viable option a valid option in good hands. It was worth mentioning that in both overall and subgroup analysis, the prognosis of open surgery was not significantly lower than that of radiotherapy and endoscopic resection. A randomized controlled trials conducted by Ogol’tsova et al. supported our result, which showed that there were no significant differences in survival between open surgery and radiotherapy (16). Clinically, the choice between endoscopic resection and open surgery in patients with early-stage GLSCC should be based on the doctor’s surgical experience and objective factors such as tumor exposure, involvement of the anterior commissure, and endoscopic safety.

Overall, there were no significant differences found in the survival outcomes among the three different studied groups. Despite the Cochrane review’s efforts to directly compare prognoses between the three groups, there was not enough evidence to support its citation and summary (15). A meta-analysis by Feng et al. demonstrated a similar result that there was no significant difference in OS and DSS between radiotherapy and endoscopic resection for the patient with early GLSCC (17). However, in subgroup analyses, differences were observed. Among cT1, N0 GLSCC patients who received endoscopic resection had higher 5-year DSS than their radiotherapy counterparts. In two recent meta-analyses, OS and DSS were significantly better with endoscopic resection than with radiotherapy, which supported our results (18, 19). Even though there was no statistically significant difference in OS or DSS between the endoscopic resection group and the radiotherapy group in the study of Huang et al, OS and DSS were skewed in favor of the endoscopic resection group (20). Differences between Huang et al.’s and our study are probably due to differences in the patient composition ratios, i.e., the patients they included had T1aN0M0, while the patients in the present study had T1N0M0. There were no differences in OS and DSS between the three treatment groups in patients ≥65 years old when analyzed as a subgroup. For patients aged <65, the prognosis was better with endoscopic resection rather than radiotherapy, likely as a result of the serious complications and long-term side effects of radiotherapy (21, 22). Few studies have been done on the treatment options for patients with early laryngeal cancer under the age of 65, and our results could provide a reference for their treatment decisions. More detailed analysis after subcategorization of patients by age and stage showed that the survival benefit of surgical treatment (either endoscopic or open) was significantly superior to radiotherapy only in the subgroup of patients aged <65 and in cT1, N0. It suggested that age and stage should be considered when selecting treatment for patients with early-stage GLSCC.

There were no significant differences in survival outcomes among patients with cT2, N0 GLSCC treated with the three treatment options, even when subgroup analysis was performed based on age. The ideal treatment for cT2, N0 GLSCC remains unclear. According to Jonathan et al. (23) combining radiotherapy and surgery is the preferred option. The American Society of Clinical Oncology (ASCO) guidelines on the treatment of laryngeal cancer (recommendation 1.2) pointed out that single-modality treatment is unlikely to be effective for deeply infiltrative glottic cancers, and so more aggressive treatments should be recommended (24). Due to the limitations of the SEER database, we were unable to confirm whether deep tumor invasion or anterior commissure involvement accounts for high tumor recurrence in cT2, N0 GLSCC after endoscopic resection. The best treatment strategy for a cT2, N0 GLSCC must be determined by prospective studies.

The major strength of the present study lies in the use of weighted scores to reduce the influence of confounding factors. Additionally, the study benefits from large sample size and a diverse population. However, this study also has some limitations. First, the lack of some useful data in the SEER database (e.g., preoperative and postoperative smoking habits, localization of the tumor, involvement of the anterior commissure or floor of the ventricle, impairment of vocal cord motility, and so on) complicated further detailed analysis. Secondly, it was a retrospective study, so there may have been unknown factors that affected the results, despite the propensity score weighting.

## Conclusion

It appears from the results of this study that age and stage should be taken into consideration when selecting treatment for patients with cT1-2, N0 GLSCC. For cT1, N0 GLSCC patients younger than 65 years of age, surgical treatment (either endoscopic or open) appears to be a preferable option over radiotherapy, in this situation, endoscopic resection may be the most appropriate option. It may be necessary for patients with cT2, N0 disease to undergo more aggressive treatment.

## Data Availability

Publicly available datasets were analyzed in this study. This data can be found here: SEER(Surveillance, Epidemiology, and End Results)-18 database;SEER account:19451-Nov2020.
