# Supplementary material for: Comparison of Survival Outcomes of Different Treatment Options for cT1-2, N0 Glottic Carcinoma: A Propensity Score–Weighted Analysis
Source: Front Surg. 2022 May 20;9:902817. doi: 10.3389/fsurg.2022.902817 (PMC9195415; doi:10.3389/fsurg.2022.902817)
Supplement: Supplementary file 1 [file Table_1_v1.docx]

Table S1. Patient characteristics in the three treatment groups before weighting

| Characteristic | Total  (N = 4274) | Endoscopic resection (n = 845)(19.8%) | Open surgery (n = 257)(6.0%) | Radiotherapy  (n = 3172)(74.2%) |
| --- | --- | --- | --- | --- |
| Race |  |  |  |  |
| White | 3647 | 741 | 233 | 2683 |
| Black | 477 | 69 | 22 | 386 |
| Other | 150 | 35 | 12 | 103 |
| Age |  |  |  |  |
| <65 | 1972 | 385 | 139 | 1448 |
| ≥65 | 2302 | 460 | 118 | 1724 |
| Sex |  |  |  |  |
| Male | 3973 | 739 | 236 | 2822 |
| Female | 477 | 106 | 21 | 350 |
| Diagnosis year |  |  |  |  |
| 2004-2009 | 2048 | 337 | 121 | 1590 |
| 2010-2015 | 2226 | 508 | 136 | 1582 |
| Marital status |  |  |  |  |
| Married | 2742 | 577 | 164 | 2001 |
| Single | 1532 | 268 | 93 | 1171 |
| Grade |  |  |  |  |
| I | 1188 | 2886 | 48 | 854 |
| II | 2658 | 488 | 172 | 1998 |
| III | 415 | 69 | 35 | 311 |
| IV | 13 | 2 | 2 | 9 |
| Stage |  |  |  |  |
| I | 3232 | 770 | 184 | 2284 |
| II | 1042 | 75 | 73 | 894 |

Table S2. Baseline characteristics of stage I patients in the three treatment groups before and after weighting

| Characteristic | Unweighted (%) |  |  |  | IPTW (%) |  |  |  |
| --- | --- | --- | --- | --- | --- | --- | --- | --- |
|  | Endoscopic resection | Open surgery | Radiotherapy | ASD | Endoscopic resection | Open surgery | Radiotherapy | ASD |
| Race |  |  |  |  |  |  |  |  |
| White | 88.4 | 88.6 | 85.1 | 0.099 | 87.1 | 88.5 | 86.2 | 0.064 |
| Black | 7.7 | 7.6 | 11.5 | 0.122 | 10.1 | 8.1 | 10.4 | 0.072 |
| Other | 3.9 | 3.8 | 3.5 | 0.023 | 2.9 | 3.4 | 3.4 | 0.029 |
| Age |  |  |  |  |  |  |  |  |
| <65 years | 44.7 | 55.4 | 45.1 | **0.216** | 45.6 | 47.0 | 45.6 | 0.029 |
| ≥65 years | 55.3 | 44.6 | 54.9 | **0.216** | 54.4 | 53.0 | 54.4 | 0.029 |
| Sex |  |  |  |  |  |  |  |  |
| Male | 87.0 | 91.3 | 88.8 | 0.137 | 88.6 | 89.8 | 88.6 | 0.038 |
| Female | 13.0 | 8.7 | 11.2 | 0.137 | 11.4 | 10.2 | 11.4 | 0.038 |
| Diagnosis year |  |  |  |  |  |  |  |  |
| 2004-2009 | 40.1 | 41.3 | 51.2 | **0.222** | 47.9 | 46.9 | 48.1 | 0.024 |
| 2010-2015 | 59.9 | 58.7 | 48.8 | **0.222** | 52.1 | 53.1 | 51.9 | 0.024 |
| Marital status |  |  |  |  |  |  |  |  |
| Married | 68.6 | 66.8 | 65.1 | 0.072 | 66 | 66.9 | 66.1 | 0.018 |
| Single | 31.4 | 33.2 | 34.9 | 0.072 | 34 | 33.1 | 33.9 | 0.018 |
| Grade |  |  |  |  |  |  |  |  |
| I | 34.7 | 17.4 | 30.0 | **0.381** | 30.5 | 29.1 | 30.3 | 0.031 |
| II | 57.1 | 69.6 | 60.4 | **0.255** | 60.5 | 62.7 | 60.3 | 0.049 |
| III | 7.9 | 12 | 9.3 | 0.138 | 8.8 | 7.9 | 9.1 | 0.041 |
| IV | 0.3 | 1.1 | 0.3 | 0.145 | 0.2 | 0.3 | 0.3 | 0.01 |

IPTW, inverse probability of treatment weighting; ASD, absolute standardized difference

ASD values in bold font indicate inadequate balance.

Table S3. Baseline characteristics of stage II patients in the three treatment groups before and after weighting

| Characteristic | Unweighted (%) |  |  |  | IPTW (%) |  |  |  |
| --- | --- | --- | --- | --- | --- | --- | --- | --- |
|  | Endoscopic resection | Open surgery | Radiotherapy | ASD | Endoscopic resection | Open surgery | Radiotherapy | ASD |
| Race |  |  |  |  |  |  |  |  |
| White | 80.0 | 82.2 | 83.3 | 0.089 | 83.7 | 83.2 | 83.2 | 0.014 |
| Black | 13.3 | 11.0 | 14.0 | 0.088 | 14.7 | 13.7 | 13.7 | 0.03 |
| Other | 6.7 | 6.8 | 2.7 | **0.244** | 1.6 | 3.1 | 3.2 | 0.093 |
| Age |  |  |  |  |  |  |  |  |
| <65 | 54.7 | 50.7 | 47.1 | 0.152 | 48.0 | 46.1 | 47.7 | 0.037 |
| ≥65 | 45.3 | 49.3 | 52.9 | 0.152 | 52.0 | 53.9 | 52.3 | 0.037 |
| Sex |  |  |  |  |  |  |  |  |
| Male | 92.0 | 93.2 | 89.5 | **0.205** | 95.4 | 96.1 | 89.9 | 0.121 |
| Female | 8.0 | 6.8 | 10.5 | **0.205** | 4.6 | 3.9 | 10.1 | 0.121 |
| Diagnosis year |  |  |  |  |  |  |  |  |
| 2004-2009 | 37.3 | 61.6 | 47.3 | **0.486** | 47.6 | 49 | 47.6 | 0.028 |
| 2010-2015 | 62.7 | 38.4 | 52.7 | **0.486** | 52.4 | 51 | 52.4 | 0.028 |
| Marital status |  |  |  |  |  |  |  |  |
| Married | 65.3 | 56.2 | 57.8 | 0.186 | 59.5 | 58.0 | 58.2 | 0.032 |
| Single | 34.7 | 43.8 | 42.2 | 0.186 | 40.5 | 42.0 | 41.8 | 0.032 |
| Grade |  |  |  |  |  |  |  |  |
| I | 25.3 | 21.9 | 19.1 | 0.157 | 19.8 | 20.3 | 19.7 | 0.015 |
| II | 64.0 | 60.3 | 69.5 | 0.198 | 72.4 | 68.5 | 68.6 | 0.084 |
| III | 10.7 | 17.8 | 11.1 | 0.223 | 7.9 | 11.2 | 11.4 | 0.112 |
| IV | 0.0 | 0.0 | 0.3 | 0.06 | 0.0 | 0.0 | 0.3 | 0.058 |

IPTW, inverse probability of treatment weighting; ASD, absolute standardized difference;

ASD values in bold font indicate inadequate balance.

Table S4. Baseline characteristics of patient aged <65 years in the three treatment groups before and after weighting

| Characteristic | Unweighted (%) |  |  |  | IPTW (%) |  |  |  |
| --- | --- | --- | --- | --- | --- | --- | --- | --- |
|  | Endoscopic resection | Open surgery | Radiotherapy | ASD | Endoscopic resection | Open surgery | Radiotherapy | ASD |
| Race |  |  |  |  |  |  |  |  |
| White | 86.5 | 88.5 | 82.1 | 0.168 | 83.6 | 89.1 | 83.5 | 0.149 |
| Black | 8.3 | 9.4 | 15.1 | 0.193 | 13.7 | 10 | 13.5 | 0.104 |
| Other | 5.2 | 2.2 | 2.8 | 0.187 | 2.7 | 0.9 | 3.1 | 0.135 |
| Sex |  |  |  |  |  |  |  |  |
| Male | 86.5 | 91.4 | 89.7 | 0.161 | 90.5 | 90.3 | 89.3 | 0.039 |
| Female | 13.5 | 8.6 | 10.3 | 0.161 | 9.5 | 9.7 | 10.7 | 0.039 |
| Diagnosis year |  |  |  |  |  |  |  |  |
| 2004-2009 | 38.2 | 51.8 | 51.4 | **0.272** | 48.4 | 49.2 | 49.0 | 0.016 |
| 2010-2015 | 61.8 | 48.2 | 48.6 | **0.272** | 51.6 | 50.8 | 51.0 | 0.016 |
| Marital status |  |  |  |  |  |  |  |  |
| Married | 66.2 | 62.6 | 63.1 | 0.076 | 63.4 | 63.8 | 63.6 | 0.008 |
| Single | 33.8 | 37.4 | 36.9 | 0.076 | 36.6 | 36.2 | 36.4 | 0.008 |
| Grade |  |  |  |  |  |  |  |  |
| I | 35.1 | 22.3 | 28.7 | **0.284** | 29.4 | 29.3 | 29.4 | 0.002 |
| II | 58.7 | 64 | 61.5 | 0.11 | 62.4 | 61.6 | 61.1 | 0.026 |
| III | 6.2 | 13.7 | 9.7 | **0.248** | 8.2 | 9.0 | 9.3 | 0.034 |
| IV | 0.0 | 0.0 | 0.2 | 0.048 | 0.0 | 0.0 | 0.2 | 0.046 |
| Stage |  |  |  |  |  |  |  |  |
| I | 89.4 | 73.4 | 70.9 | **0.407** | 76.1 | 75.9 | 74.6 | 0.032 |
| II | 10.6 | 26.6 | 29.1 | **0.407** | 23.9 | 24.1 | 25.4 | 0.032 |

IPTW, inverse probability of treatment weighting; ASD, absolute standardized difference

ASD values in bold font indicate inadequate balance.

Table S5. Baseline characteristics of patient aged ≥65 years in the three treatment groups before and after weighting

| Characteristic | Unweighted (%) |  |  |  | IPTW (%) |  |  |  |
| --- | --- | --- | --- | --- | --- | --- | --- | --- |
|  | Endoscopic resection | Open surgery | Radiotherapy | ASD | Endoscopic resection | Open surgery | Radiotherapy | ASD |
| Race |  |  |  |  |  |  |  |  |
| White | 88.7 | 84.7 | 86.7 | 0.116 | 87.2 | 89.4 | 87.1 | 0.068 |
| Black | 8.0 | 7.6 | 9.7 | 0.07 | 9.6 | 6.7 | 9.3 | 0.098 |
| Other | 3.3 | 7.6 | 3.7 | **0.225** | 3.2 | 3.9 | 3.6 | 0.036 |
| Sex |  |  |  |  |  |  |  |  |
| Male | 88.3 | 92.4 | 88.3 | 0.129 | 88.6 | 90.7 | 88.6 | 0.065 |
| Female | 11.7 | 7.6 | 11.7 | 0.129 | 11.4 | 9.3 | 11.4 | 0.065 |
| Diagnosis year |  |  |  |  |  |  |  |  |
| 2004-2009 | 41.3 | 41.5 | 49.1 | 0.155 | 46.8 | 44.5 | 47.2 | 0.055 |
| 2010-2015 | 58.7 | 58.5 | 50.9 | 0.155 | 53.2 | 55.5 | 52.8 | 0.055 |
| Marital status |  |  |  |  |  |  |  |  |
| Married | 70.0 | 65.3 | 63.1 | 0.144 | 65.7 | 66.3 | 64.5 | 0.037 |
| Single | 30.0 | 34.7 | 36.9 | 0.144 | 34.3 | 33.7 | 35.5 | 0.037 |
| Grade |  |  |  |  |  |  |  |  |
| I | 32.8 | 14.4 | 25.5 | **0.427** | 26.8 | 23.8 | 26.3 | 0.069 |
| II | 57.0 | 70.3 | 64.3 | 0.28 | 63.7 | 66.1 | 63.3 | 0.058 |
| III | 9.8 | 13.6 | 9.9 | 0.125 | 9.2 | 9.7 | 10 | 0.028 |
| IV | 0.4 | 1.7 | 0.3 | **0.205** | 0.3 | 0.4 | 0.4 | 0.013 |
| Stage |  |  |  |  |  |  |  |  |
| I | 92.6 | 69.5 | 72.6 | **0.517** | 78.0 | 76.2 | 76.4 | 0.038 |
| II | 7.4 | 30.5 | 27.4 | **0.517** | 22.0 | 23.8 | 23.6 | 0.038 |

IPTW, inverse probability of treatment weighting; ASD, absolute standardized difference

ASD values in bold font indicate inadequate balance.Table S6. Baseline characteristics of stage I patient aged <65 years in the three treatment groups before and after weighting

| Characteristic | Unweighted (%) |  |  |  | IPTW (%) |  |  |  |
| --- | --- | --- | --- | --- | --- | --- | --- | --- |
|  | Endoscopic resection | Open surgery | Radiotherapy | ASD | Endoscopic resection | Open surgery | Radiotherapy | ASD |
| Race |  |  |  |  |  |  |  |  |
| White | 88.1 | 89.2 | 82.9 | 0.171 | 85.1 | 88.1 | 84.7 | 0.093 |
| Black | 7.0 | 9.8 | 14.3 | **0.212** | 12.1 | 11.2 | 12.3 | 0.031 |
| Other | 4.9 | 1.0 | 2.8 | **0.246** | 2.8 | 0.6 | 3.0 | 0.148 |
| Sex |  |  |  |  |  |  |  |  |
| Male | 85.5 | 92.2 | 89.8 | **0.223** | 89.1 | 90.0 | 89.1 | 0.029 |
| Female | 14.5 | 7.8 | 10.2 | **0.223** | 10.9 | 10.0 | 10.9 | 0.029 |
| Diagnosis year |  |  |  |  |  |  |  |  |
| 2004-2009 | 38.1 | 46.1 | 52.6 | **0.290** | 48.7 | 49.5 | 48.9 | 0.016 |
| 2010-2015 | 61.9 | 53.9 | 47.4 | **0.290** | 51.3 | 50.5 | 51.1 | 0.016 |
| Marital status |  |  |  |  |  |  |  |  |
| Married | 67.4 | 63.7 | 65.0 | 0.078 | 65.2 | 65.7 | 65.6 | 0.01 |
| Single | 32.6 | 36.3 | 35.0 | 0.078 | 34.8 | 34.3 | 34.4 | 0.01 |
| Grade |  |  |  |  |  |  |  |  |
| I | 35.8 | 19.6 | 31.7 | 0.35 | 31.9 | 31.8 | 31.7 | 0.003 |
| II | 58.1 | 68.6 | 58.8 | **0.214** | 59.5 | 60.5 | 59.4 | 0.023 |
| III | 6.1 | 11.8 | 9.2 | 0.194 | 8.6 | 7.6 | 8.6 | 0.033 |
| IV | 0.0 | 0.0 | 0.3 | 0.057 | 0.0 | 0.0 | 0.3 | 0.052 |

IPTW, inverse probability of treatment weighting; ASD, absolute standardized difference

ASD values in bold font indicate inadequate balance.

Table S7. Baseline characteristics of stage I patients aged ≥65 years in the three treatment groups before and after weighting

| Characteristic | Unweighted (%) |  |  |  | IPTW (%) |  |  |  |
| --- | --- | --- | --- | --- | --- | --- | --- | --- |
|  | Endoscopic resection | Open surgery | Radiotherapy | ASD | Endoscopic resection | Open surgery | Radiotherapy | ASD |
| Race |  |  |  |  |  |  |  |  |
| White | 88.7 | 87.8 | 86.9 | 0.055 | 88.0 | 89.6 | 87.6 | 0.059 |
| Black | 8.2 | 4.9 | 9.1 | 0.149 | 8.6 | 6.7 | 8.7 | 0.071 |
| Other | 3.1 | 7.3 | 4.0 | **0.213** | 3.3 | 3.7 | 3.7 | 0.017 |
| Sex |  |  |  |  |  |  |  |  |
| Male | 88.3 | 90.2 | 87.9 | 0.071 | 88.2 | 89.6 | 88.1 | 0.045 |
| Female | 11.7 | 9.8 | 12.1 | 0.071 | 11.8 | 10.4 | 11.9 | 0.045 |
| Diagnosis year |  |  |  |  |  |  |  |  |
| 2004-2009 | 41.8 | 35.4 | 50.1 | **0.295** | 47.3 | 45.3 | 47.6 | 0.045 |
| 2010-2015 | 58.2 | 64.6 | 49.9 | **0.295** | 52.7 | 54.7 | 52.4 | 0.045 |
| Marital status |  |  |  |  |  |  |  |  |
| Married | 69.5 | 70.7 | 65.2 | 0.116 | 66.7 | 66.6 | 66.4 | 0.005 |
| Single | 30.5 | 29.3 | 34.8 | 0.116 | 33.3 | 33.4 | 33.6 | 0.005 |
| Grade |  |  |  |  |  |  |  |  |
| I | 33.8 | 14.6 | 28.5 | **0.428** | 29.0 | 26.7 | 29 | 0.053 |
| II | 56.3 | 70.7 | 61.8 | **0.297** | 61.0 | 62.9 | 61.2 | 0.038 |
| III | 9.4 | 12.2 | 9.4 | 0.095 | 9.5 | 9.0 | 9.6 | 0.018 |
| IV | 0.5 | 2.4 | 0.2 | **0.360** | 0.5 | 1.4 | 0.2 | 0.187 |

IPTW, inverse probability of treatment weighting; ASD, absolute standardized difference

ASD values in bold font indicate inadequate balance.

Table S8. Baseline characteristics of stage II patients aged <65 years in the three treatment groups before and after weighting

| Characteristic | Unweighted (%) |  |  |  | IPTW (%) |  |  |  |
| --- | --- | --- | --- | --- | --- | --- | --- | --- |
|  | Endoscopic resection | Open surgery | Radiotherapy | ASD | Endoscopic resection | Open surgery | Radiotherapy | ASD |
| Race |  |  |  |  |  |  |  |  |
| White | 73.2 | 86.5 | 80.3 | **0.338** | 79.1 | 85.8 | 80.1 | 0.168 |
| Black | 19.5 | 8.1 | 17.1 | **0.308** | 18.6 | 11.7 | 16.7 | 0.188 |
| Other | 7.3 | 5.4 | 2.6 | **0.283** | 2.2 | 2.6 | 3.2 | 0.058 |
| Sex |  |  |  |  |  |  |  |  |
| Male | 95.1 | 89.2 | 89.5 | 0.194 | 95.2 | 93.7 | 89.9 | 0.172 |
| Female | 4.9 | 10.8 | 10.5 | 0.194 | 4.8 | 6.3 | 10.1 | 0.172 |
| Diagnosis year |  |  |  |  |  |  |  |  |
| 2004-2009 | 39.0 | 67.6 | 48.5 | **0.571** | 47.9 | 49.2 | 48.9 | 0.026 |
| 2010-2015 | 61.0 | 32.4 | 51.5 | **0.571** | 52.1 | 50.8 | 51.1 | 0.026 |
| Marital status |  |  |  |  |  |  |  |  |
| Married | 56.1 | 59.5 | 58.4 | 0.068 | 56.6 | 56.7 | 58.1 | 0.032 |
| Single | 43.9 | 40.5 | 41.6 | 0.068 | 43.4 | 43.3 | 41.9 | 0.032 |
| Grade |  |  |  |  |  |  |  |  |
| I | 29.3 | 29.7 | 21.1 | **0.208** | 22.6 | 23.2 | 22.4 | 0.019 |
| II | 63.4 | 51.4 | 67.9 | **0.352** | 71.7 | 66.3 | 66.4 | 0.114 |
| III | 7.3 | 18.9 | 10.9 | **0.363** | 5.7 | 10.5 | 11.1 | 0.172 |
| IV | 0.0 | 0.0 | 0.1 | 0.080 | 0.0 | 0.0 | 0.1 | 0.081 |

IPTW, inverse probability of treatment weighting; ASD, absolute standardized difference

ASD values in bold font indicate inadequate balance.

Table S9. Baseline characteristics of stage II patients aged ≥65 years in the three treatment groups before and after weighting

| Characteristic | Unweighted (%) |  |  |  | IPTW (%) |  |  |  |
| --- | --- | --- | --- | --- | --- | --- | --- | --- |
|  | Endoscopic resection | Open surgery | Radiotherapy | ASD | Endoscopic resection | Open surgery | Radiotherapy | ASD |
| Race |  |  |  |  |  |  |  |  |
| White | 88.2 | 77.8 | 86 | **0.297** | 86.1 | 85.7 | 85.7 | 0.011 |
| Black | 5.9 | 13.9 | 11.2 | **0.252** | 11.7 | 11.1 | 11.1 | 0.02 |
| Other | 5.9 | 8.3 | 2.7 | **0.320** | 2.2 | 3.1 | 3.2 | 0.058 |
| Sex |  |  |  |  |  |  |  |  |
| Male | 88.2 | 97.2 | 89.4 | **0.299** | 90.3 | 97.8 | 89.8 | **0.266** |
| Female | 11.8 | 2.8 | 10.6 | **0.299** | 9.7 | 2.2 | 10.2 | **0.266** |
| Diagnosis year |  |  |  |  |  |  |  |  |
| 2004-2009 | 35.3 | 55.6 | 46.3 | **0.406** | 43.7 | 47.6 | 46.4 | 0.079 |
| 2010-2015 | 64.7 | 44.4 | 53.7 | **0.406** | 56.3 | 52.4 | 53.6 | 0.079 |
| Married status |  |  |  |  |  |  |  |  |
| Married | 76.5 | 52.8 | 57.3 | **0.479** | 67.2 | 58.3 | 58.1 | 0.182 |
| Single | 23.5 | 47.2 | 42.7 | **0.479** | 32.8 | 41.7 | 41.9 | 0.182 |
| Grade |  |  |  |  |  |  |  |  |
| I | 20.6 | 13.9 | 17.3 | 0.178 | 17.2 | 16.6 | 17.3 | 0.018 |
| II | 64.7 | 69.4 | 70.8 | 0.134 | 73.3 | 71 | 70.5 | 0.062 |
| III | 14.7 | 16.7 | 11.2 | 0.171 | 9.5 | 12.4 | 11.6 | 0.092 |
| IV | 0.0 | 0.0 | 0.6 | 0.083 | 0.0 | 0.0 | 0.6 | 0.08 |

IPTW, inverse probability of treatment weighting; ASD, absolute standardized difference

ASD values in bold font indicate inadequate balance.


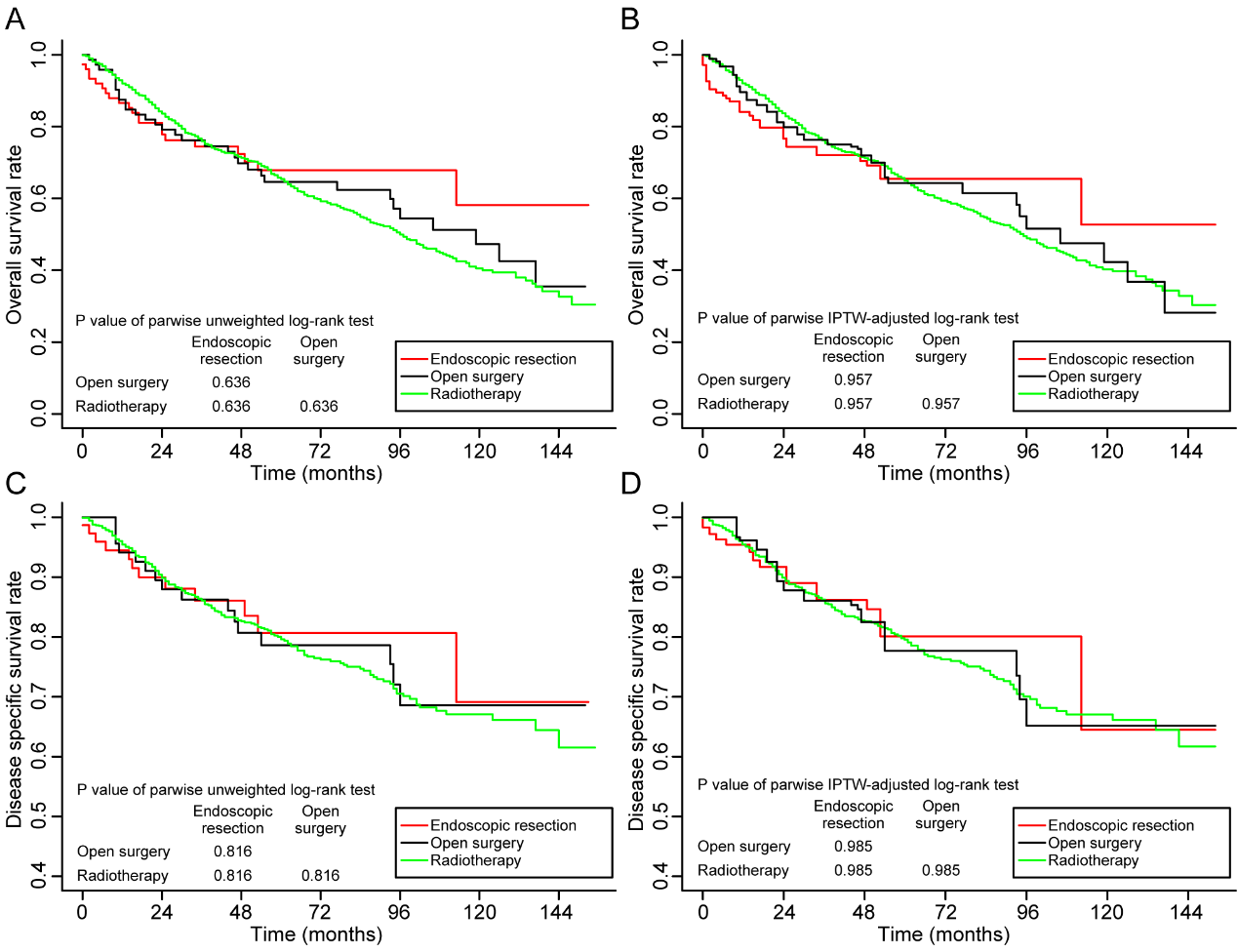


Figure S1. Kaplan–Meier curves for overall survival (A, B) and disease-specific survival (C, D) before and after weighting for stage II patients. The p values in bold font are statistically significant.

IPTW, inverse probability of treatment weighting.


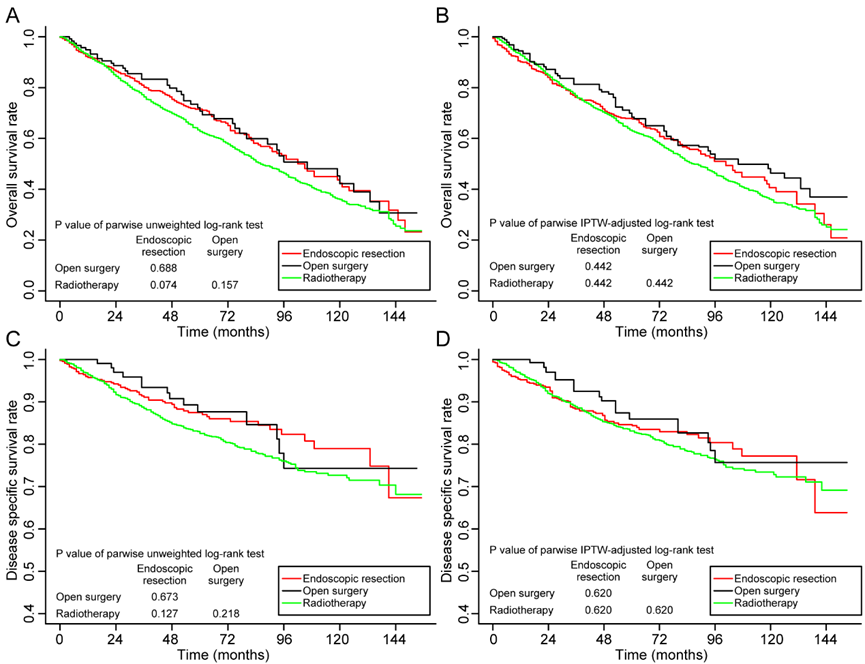


Figure S2. Kaplan–Meier curves for overall survival (A, B) and disease-specific survival (C, D) before and after weighting for patients aged ≥65 years. The p values in bold font are statistically significant.

IPTW, inverse probability of treatment weighting.


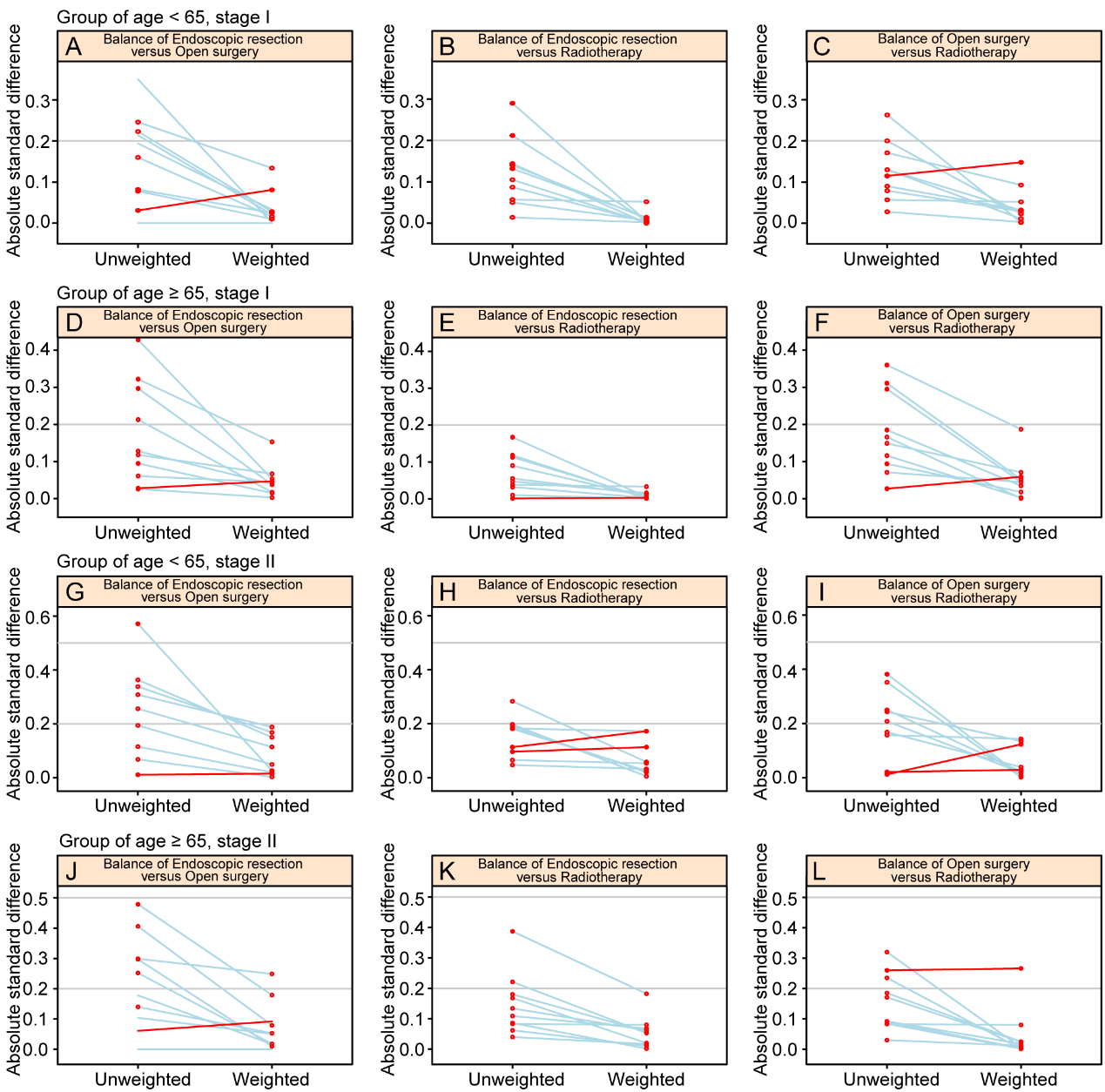


Figure S3. Paired graphs showing the balance of baseline characteristics among treatment groups for stage I patients aged <65 years (A–C), stage I patients aged ≥65 years (D–F), stage II patients aged <65 years (G–I), and stage II patients aged ≥65 years (J–L).


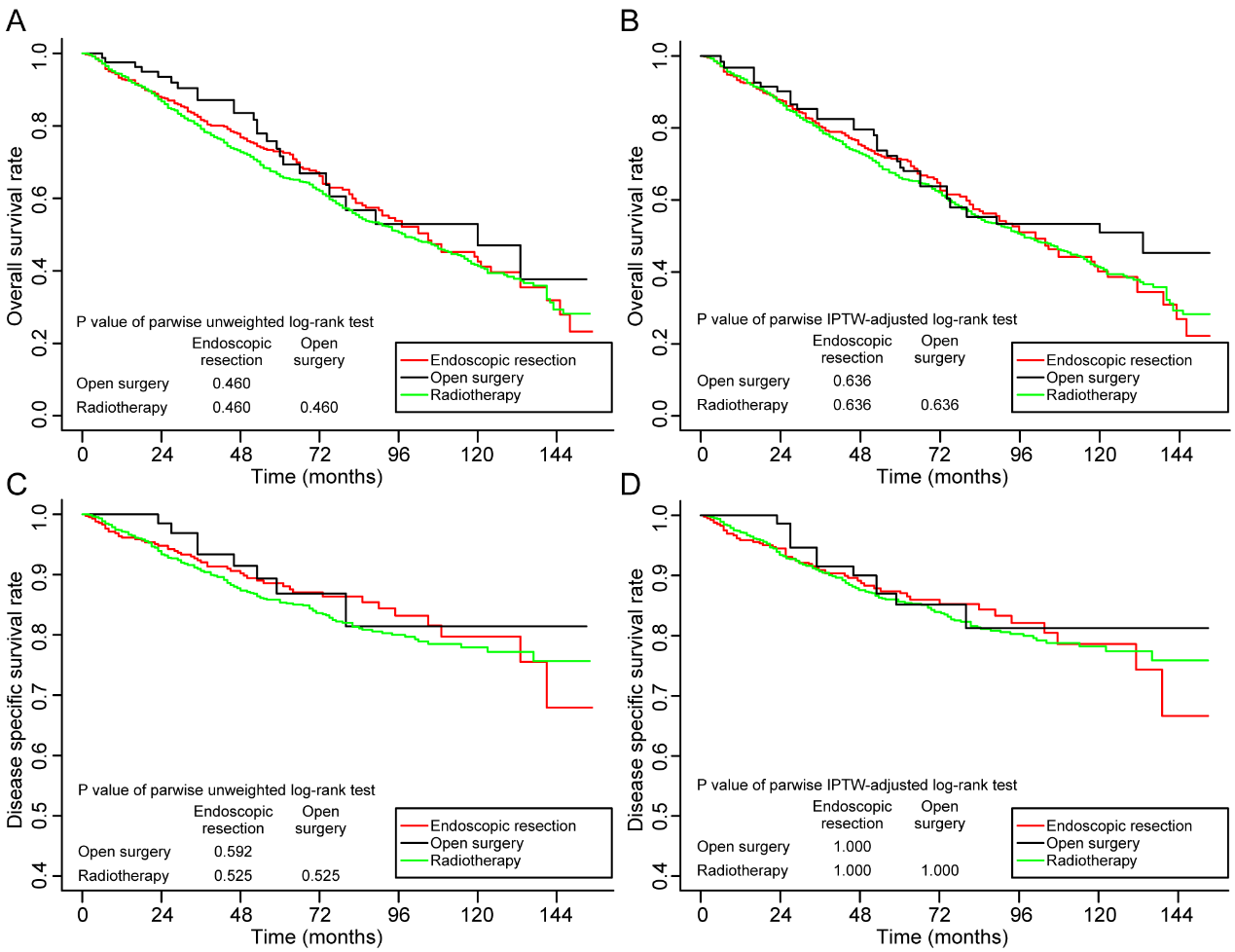


Figure S4. Kaplan–Meier curves for overall survival (A, B) and disease-specific survival (C, D) before and after weighting for stage I patients aged ≥65 years. The p values in bold font are statistically significant.

IPTW, inverse probability of treatment weighting.


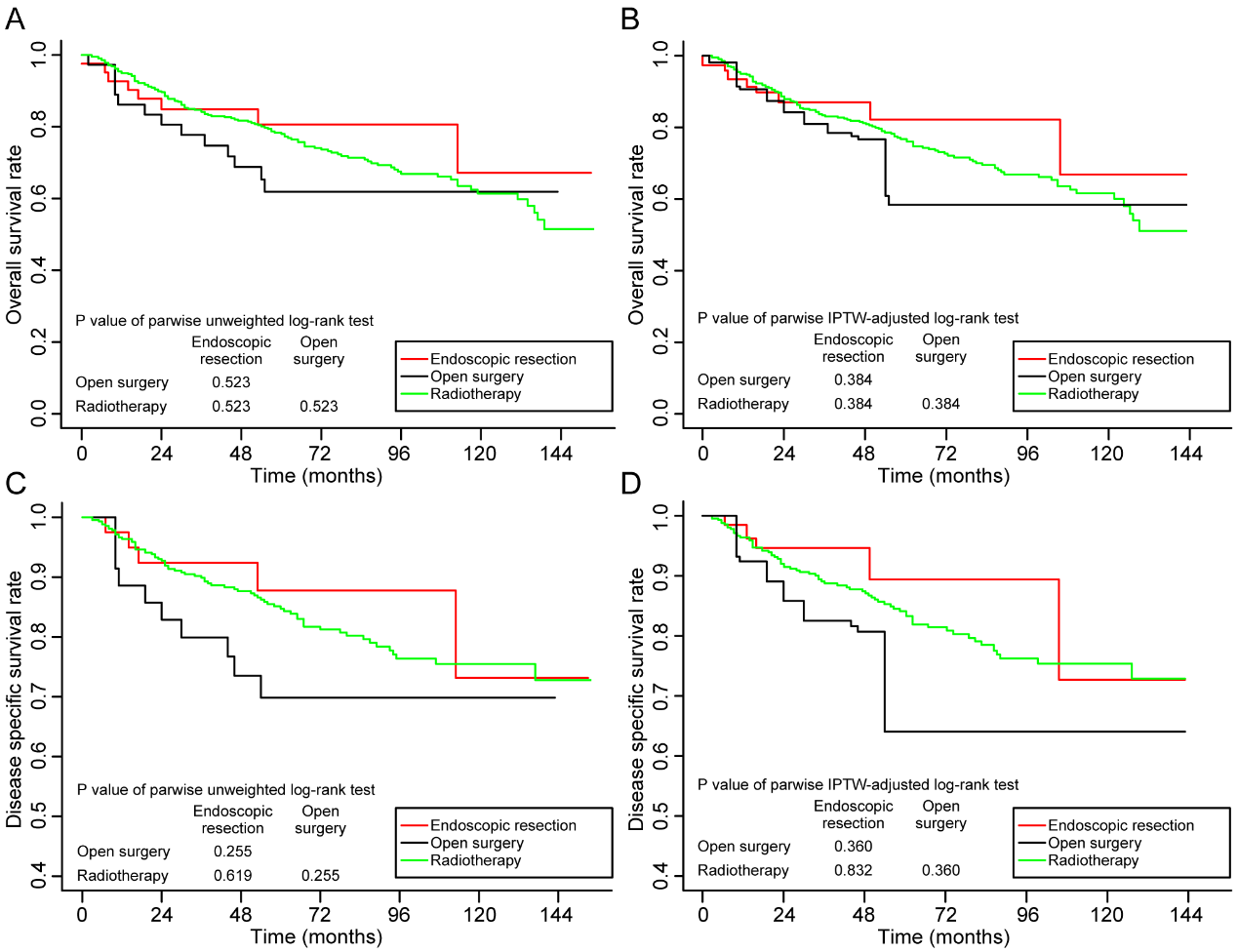


Figure S5. Kaplan–Meier curves for overall survival (A, B) and disease-specific survival (C, D) before and after weighting for stage II patients aged <65 years. The p values in bold font are statistically significant.

IPTW, inverse probability of treatment weighting.


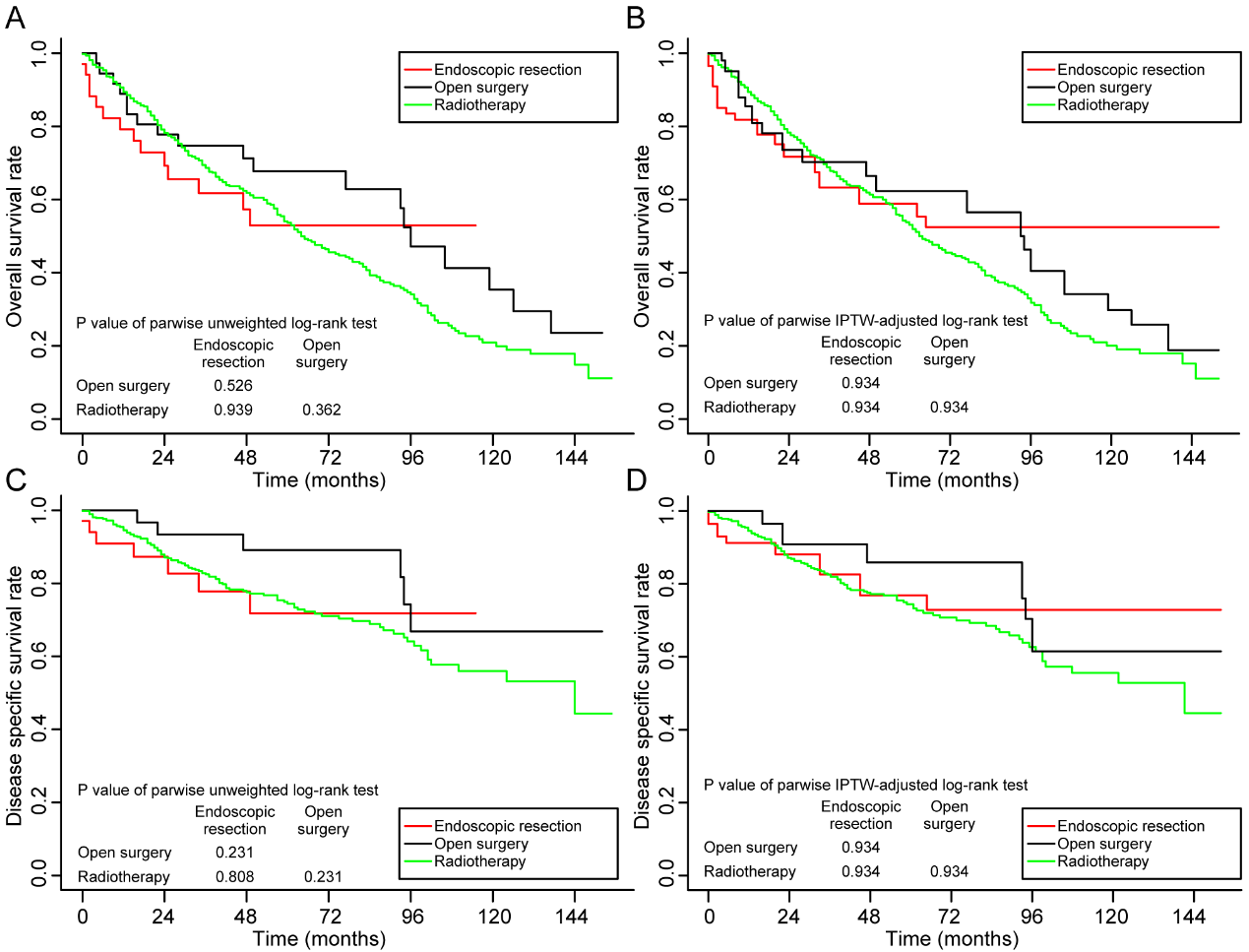


Figure S6. Kaplan–Meier curves for overall survival (A, B) and disease-specific survival (C, D) before and after weighting for stage II patients aged ≥65 years. The p values in bold font are statistically significant.

IPTW, inverse probability of treatment weighting.
